# Correcting positional correlations in Affymetrix® Genome Chips

**DOI:** 10.1038/srep09078

**Published:** 2015-03-13

**Authors:** Dirar Homouz, Gang Chen, Andrzej S. Kudlicki

**Affiliations:** 1 Khalifa University of Science, Technology and Research, Abu Dhabi, UAE; 2 BGI Tech, Shenzhen, China; 3 Department of Biochemistry and Molecular Biology, University of Texas Medical Branch, Galveston, TX, USA; 4 Institute for Translational Sciences, University of Texas Medical Branch, Galveston, TX, USA; 5 Sealy Center for Molecular Medicine, University of Texas Medical Branch, Galveston, TX, USA

## Abstract

We report and model a previously undescribed systematic error causing spurious excess correlations that depend on the distance between probes on Affymetrix® microarrays. The phenomenon affects pairs of features with large chip separations, up to over 100 probes apart. The effect may have a significant impact on analysis of correlations in large collections of expression data, where the systematic experimental errors are repeated in many data sets. Examples of such studies include analysis of functions and interactions in groups of genes, as well as global properties of genomes. We find that the average correlations between probes on Affymetrix microarrays are larger for smaller chip distances, which points out to a previously undescribed positional artifact. The magnitude of the artifact depends on the design of the chip, and we find it to be especially high for the yeast S98 microarray, where spurious excess correlations reach 0.1 at a distance of 50 probes. We have designed an algorithm to correct this bias and provide new data sets with the corrected expression values. This algorithm was successfully implemented to remove the positional artifact from the S98 chip data while preserving the integrity of the data.

## Overview

DNA microarrays provide a means of measuring the concentrations of thousands of genetic sequences in one experiment, and are used in many fields ranging from basic biological research to medical applications^1^,^2^,^3^,^4^,^5^,^6^,^7^. In gene expression profiling^8^,^9^,^10^,^11^,^12^ experiments, microarrays are used to measure the mRNA concentration for large numbers of genes.

This technology has produced thousands of experimental data sets that are available through online databases such as the Gene Expression Omnibus (GEO)^13^. While expression profiling is now often done using RNA-seq techniques, large collections of legacy microarray data are often analyzed to reveal functional relationships between coexpressed genes, as well as to infer regulatory interactions, e.g.^14^,^15^,^16^,^17^,^18^,^19^,^20^. Inference from large datasets is especially prone to systematic errors that might be repeated in every experiment. In the widely used Affymetrix GeneChip platforms, several kinds of systematic errors have been reported, such as the order-dependence of expression values^21^, correlated expression between probes containing runs of guanine^22^ and image artifacts that enhance outliers^23^. In spotted microarrays, the systematic errors include positional effects that lead to spurious correlations between the measured expression levels of the probes on the microarray^24^,^25^,^26^. This positional artifact in the spotted arrays has been attributed to carryover during the transfer of presynthesized probes onto the chip^24^.

## Position effect in Affymetrix® chips

In this paper we consider the Affymetrix microarray platforms, specifically the Yeast Genome S98 Array, a popular whole genome platform for *Saccharomyces cerevisiae*. In order to investigate systematic position effects, we analyzed the expression data available in the Gene Expression Omnibus database. We observed that the data collected for experiments done using the S98 chip display long-range excess correlations strongly dependent on the distance between the probes on the chip. Although the effects are similar in appearance to the ones observed in spotted microarrays, the physical nature of the positional artifact in Affymetrix chips must be different because the Affymetrix chips are fabricated using *in situ* synthesis of the probes.

Systematic excess correlations may bias the inference of functional gene relationships from large numbers of experiments. To describe and correct these effects, we modeled this artifact in S98 chips and designed an algorithm to correct for it by reconstructing the unbiased expression values. Our model is derived based on a large collection of array data and allows the removal of spurious correlations from any set of experiments that use the same chip.

## Results

### Evidence of positional artifact

The Affymetrix Yeast Genome S98 Array contains 9335 probe sets with a total of 285,156 probes. To characterize the dependence of the correlations between different probe sets on their physical location on the chip, we computed a map of average correlation as a function of probe separation along the x-axis (dx) and along the y-axis (dy). Every pixel in this correlation map represents the average of all pairs of probes with a given separation on the chip (with dimensions *L* and *H*); see definition in Methods below. Therefore, if no positional effects were present, one would expect this correlation map to be flat and only random oscillations around the average correlation in the genome would be observed; a small positive number, depending on the collection of experiments used in the analysis.

**Figure 1:**
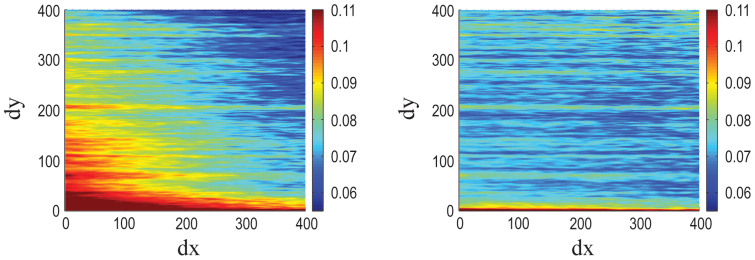
Original S98 Array correlation map compared to the corrected one. The two dimensional map of the average correlation between two different probes on the Affymetrix Yeast Genome S98 Array as a function of the separation (*dx*,*dy*) for the original data (a) and the corrected data (b). The separation between two neighboring probes (24 μm) is used as a unit of distance.

**Figure 2:**
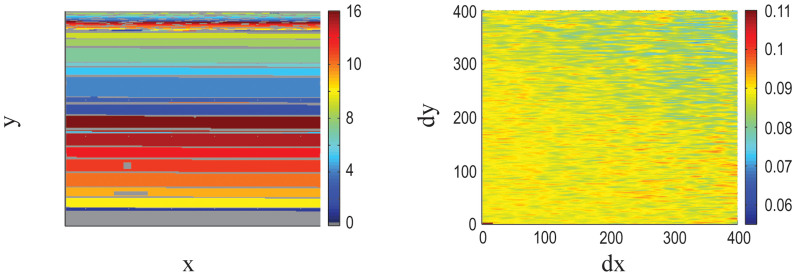
(a) The layout of probes with targets from the different 16 Yeast chromosomes. Each color band represents probes for one chromosome. (b) The two dimensional map of the average correlation between two different probes on the Affymetrix Yeast Genome S98 Chip as a function of the separation (*dx*,*dy*) for a simulated data with probes at randomized positions.

The actual correlation map, created from 1383 samples is shown in Figure 1a. One prominent non-random feature is the stronger correlation between probes along the x-axis. This effect can be explained by the fact that the probes with genes from the same chromosome are typically laid out next to each other along the x-axis of the chip. Figure 2a shows these bands of probes with the same chromosomal targets. This kind of arrangement increases correlations between probes along the x-axis since neighboring genes on one chromosome are more likely to have the same expression pattern; they often share the same transcription factor binding sites^27^,^28^.

The second prominent feature in the map in Figure 1a is that the correlation between two probes is strongly dependent on their Euclidean chip distance. Probes with smaller separation on the chip tend to correlate more than probes with large chip separation, regardless of chromosomal proximity; this effect extends to distances in excess of 100 probes. Such a specific pattern on the chip is unlikely to have a biological cause and we propose that it results from experimental systematic errors. To demonstrate this, we recalculated the correlation map for a simulated chip with probes shuffled into random positions. The correlation coefficient between any two different probe sets in the randomized chip was the same as in the original configuration, but the average correlation map for the simulated microarray was not dependent on chip distance (see Figure 2b).

The positional bias is also present in other Affymetrix platforms for yeast and other organisms. We confirmed it in the Yeast2 Array as well as in two mouse platforms: Mouse Expression Array 430A and Mouse Expression Array 430B. However, the artifact has the greatest impact on interpretation of the correlations in the data in the Yeast S98 Array owing to the particular design of this chip: probes to the same gene are typically placed next to each other along the x-axis of the chip. The spurious correlations in Yeast2, 430A, and 430B arrays are an order of magnitude lower than in S98.

Further confirmation of the presence of the positional artifact, independent of the correlation analysis, comes from studying the mismatch probes on the microarray. Since the intensities measured in the mismatch (MM) probes are typically lower than in the perfect match (PM) probes, the effect is expected to result in PM signal overflowing into the MM probes in their vicinity.

**Figure 3:**
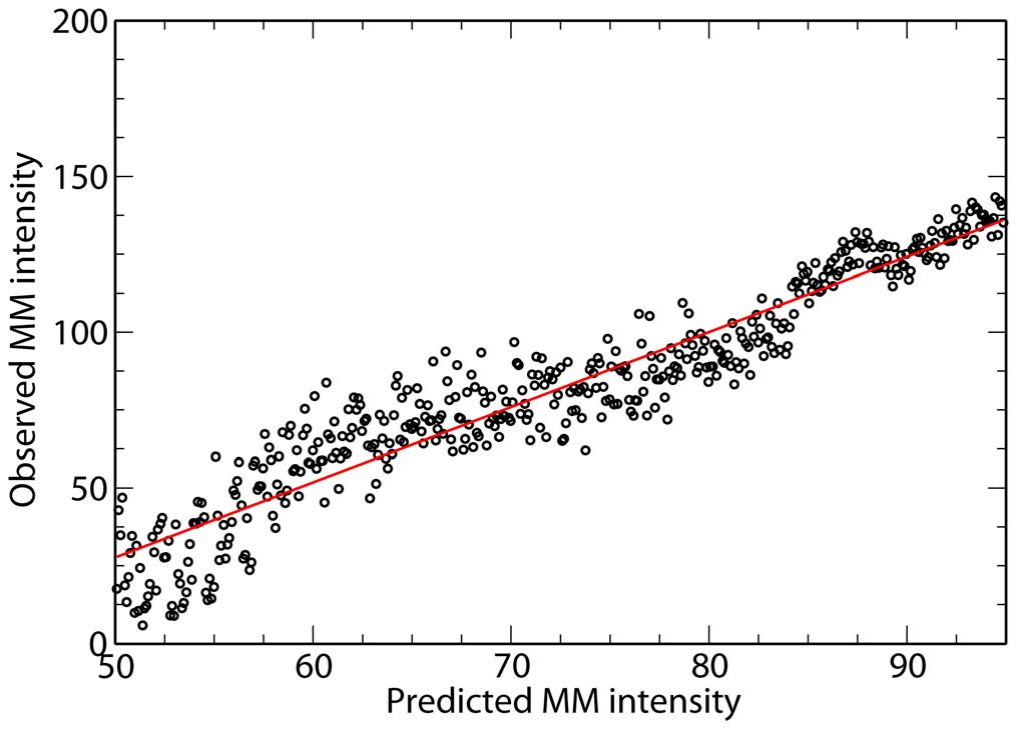
The positional artifact contributes to the mismatch probes on the Yeast2 array. The relation between the signal in the mismatch probes: expected from the position artifact vs. observed. The data are divided into bins according to the predicted value, and the median measured signal is shown in each of the bins.

We measured this phenomenon using data from the Yeast2 microarray. Based on our model of the artifact, we predicted the intensity of the spurious signal in each of the mismatch probes, and compared it with the measured MM signal. We binned the observed MM intensities vs. the predicted ones in the logarithmic scale and computed the average in each respective bin. The correlation coefficient between predicted spurious signal and the average MM measurement is 0.949, with a p-value of 2.5e-7 (see Figure 3). This not only proves that the positional bias does exist in the Yeast2 array, but also demonstrates that it is responsible for a significant fraction of the signal in the mismatch probes.

### Modeling and correcting the artifact

The positional artifact in the microarray data extends over large distances on the chip, which means that it originates via a long-range process. Elucidating the physical mechanism behind this effect is beyond the scope of this paper, but possibilities include the diffusion of fluorophores during the washing stage of the experiment, diffusion of light while scanning the array, or a combination thereof. In spotted microarrays, several methods have been suggested to correct for similar positional artifacts, either by correcting the expression values for all probes^29^, or for the predicted correlations^25^. The latter method concentrates on correcting correlations without justifying the effect of these corrections on the expression values.

Here, we modeled the effect as partial diffusion of the signal in two dimensions. Based on this assumption, the original, unaffected expression values can be reconstructed from the data by using a deconvolution procedure with a two-component model kernel designed to remove any possible diffusive effects (see Methods for detailed discussion). To this end, we implemented an algorithm for optimizing the shape of the model kernel to best reconstruct the true expression values, and used the corrected expression values to compute the corrected correlation chip map, Figure 1b. Our model estimates that approximately 26% of the signal gets diffused to surrounding pixels. In the corrected data, we still observe the stronger correlation along the x-axis but the positional artifact was removed to a large extent (90% removed at a distance of 50 probes). This map is now similar to the map generated for the randomized data, (Figure 2b), with the exception that the average correlations in the corrected maps are lower. Figure 2a shows that the correlation coefficient depends on the radial chip distance for the original, randomized, as well as the deconvolved (corrected) data.

Creating special software to model and remove the effect has been necessary because existing preprocessing packages (like Bioconductor^30^) do not include a procedure for modeling and correcting such position artifacts. Also, our analysis shows that the positional artifact persists in the S98 array even after applying popular processing packages such as MAS, RMA, GCRMA, or DChiP.

**Figure 4:**
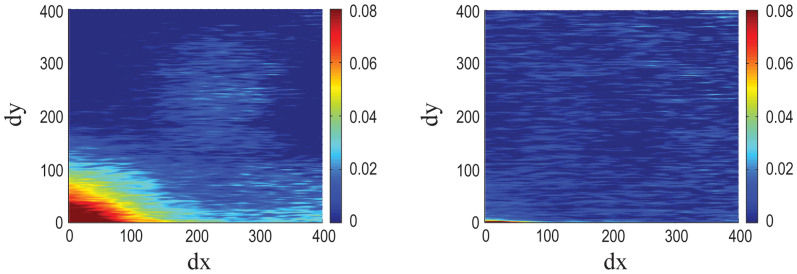
The position effect in a single experiment. The two dimensional map of the average correlation between two different probes on the Affymetrix Yeast Genome S98 Chip as a function of the separation (*dx*,*dy*) for the original Yeast Metabolic Cycle data (a) and the corrected data (b).

To demonstrate how our method improves smaller collections of data, we computed the correlation map for a single series of experiments; the Yeast Metabolic Cycle (YMC)^31^. The results are shown in Figure 4. Again, deconvolving the observed data with our optimized kernel did indeed remove the positional bias. This also shows that the method can be applied to any new experiment done using the S98 chip.

**Figure 5:**
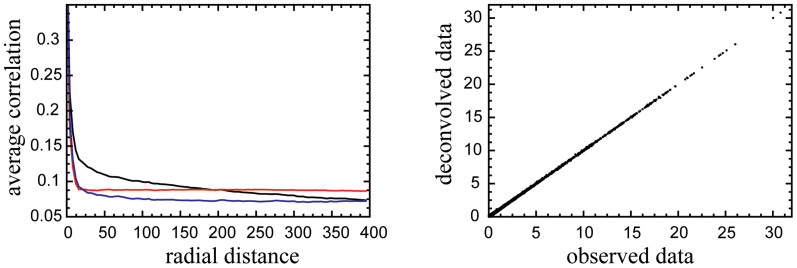
Comparison of the average correlation and the expression values. (a) The average correlation between two probes on the Affymetrix Yeast Genome S98 Array as a function of the radial distance between the two probes for the original data (black), probes with randomized positions (red) and deconvolved data (blue). (b) Comparison between the original expression levels, the x-axis, and the deconvolved data, the y-axis, for one sample, (GEO accession number: GSM6711).

Unlike other deconvolution methods previously used^24^,^25^,^29^, our correction method is designed in such a way that it removes the positional artifact while preserving the integrity of the original data. Figure 5 shows a typical relationship between the original and corrected data for one of the samples. The figure shows that the required corrections in the expression are generally small. Again these characteristics of the correction method hold true for any number of experimental samples.

### Significance of the correction

**Figure 6:**
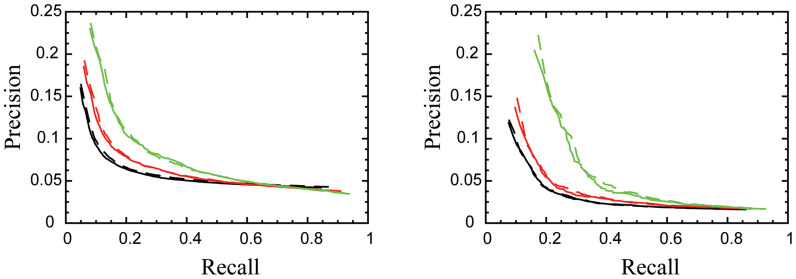
The precision-recall curves for the assignment of TF target genes. The precision-recall curves for the assignment of target genes of multiple Yeast transcription factors based on the YEASTRACT database (a) and the work of Harbison *et al.* (b). The precision-recall curves for the original Affymetrix data are shown in solid lines and the deconvolved data in dashed lines. The curves are based on the average of all transcription factors (black), the transcription factors with tightest cluster of targets, top 50%, (red), and top 25% (green).

The positional artifact in the microarray data may influence the interpretation of results based on the analysis of a large number of genes such as gene annotation or network inference. In such studies the repeated systematic effects become significant even at low magnitude. In order to test the effects of these corrections on our understanding of gene regulation, we analyzed the expression of target genes of transcription factors (TFs) in *Saccharomyces cerevisiae*. The targets of many TFs are expected to share a mode of regulation and they exhibit correlations in their expression profiles. There are several published lists of Yeast TF's with their targets. The assignments of the targets of TF in these are based on different experimental and computational techniques. Here we considered two different sets of data, YEASTRACT^32^, and the work of Harbison et al.^33^. We tested whether the expected co-expression of TF targets was more prominent in the corrected or the original data. The targets of a TF in these two sets (YEASTRACT and Harbison) were determined using different query methods and experimental techniques independent of the microarray expression data, which is important in our testing to avoid circular reasoning. For each transcription factor, we defined the centroid of the cluster of its targets as the target with the highest average correlation with all other members in the cluster. Next, we ranked the genes based on their strength of correlation with the centroid of the cluster. This ranking algorithm was used to assess coexpression of genes before and after applying the deconvolution correction. The results are summarized in Figure 6, which shows the precision-recall (PR) curves of this algorithm for all TFs, as well as for the sets of 50% and 25% TFs with the most tightly correlated targets. In each case, the solid line represents the PR curve from uncorrected data while the dashed ones show the PR curve from the corrected data. This comparison indicates that coexpression in the corrected data is slightly but consistently improved over the original data. The improvement is stronger in the tighter clusters than in the entire collections of TFs, and it is also more prominent in the YEASTRACT than in the Harbison sets.

## Discussion

The analysis of hundreds of expression microarray experiments using the Affymetrix S98 yeast genome chip has led us to uncover a systematic error that manifests in the form of excess correlations between nearby probes on the chip. These spurious correlations depend strongly on the distance between probes. This positional artifact may interfere with interpretation of whole-genome gene expression studies. Removing this artifact is a necessary step in inferring gene relationships from large-scale data sets. We designed a computational method that removes the spurious correlations by correcting the measured expression levels. Our method is based on the assumption that the effect is consistent with a diffusion effect that may be taking place either during the washing or the scanning stage of a microarray experiment. Thus, the measured expression level of each probe is convolved by contributions from the diffused signal. We reconstructed the true expression levels by applying deconvolution with a model kernel, and show that this successfully removes the positional bias. This method was implemented using the CUDA GPU computing technology, which allowed a 480-fold speed up of computations. The corrected data are provided online at http://moment.utmb.edu/S98/. Our model kernel can be used with no change to correct the expression values in any experiment done with the S98 platform. The computer program for performing the deconvolution is also available on the website. Similar artifacts are also present in other Affymetrix platforms, including the Yeast2 array and the mouse arrays MOE430A and MOE430B. However, the way this artifact manifests depends strongly on the design of the array, especially on the relative positions of probes within a probe set. Specifically, the excess correlations in the Yeast2, MOE430A and MOE430B arrays are an order of magnitude lower than in the yeast S98 microarrays. We expect that our approach, with little or no modification, can detect, quantify and correct positional artifacts in different platforms. In the future we intend to investigate the underlying physical mechanisms of this positional artifact.

## Methods

### Source of data

The primary sources of data for this work are the microarray datasets available from the GEO website for the Affymetrix Yeast Genome S98 Array. To describe and parameterize the positional effect, we utilized 1383 available data sets in the GPL90 platform family (the list is available on the supporting webpage). We used the probe set data according to the GPL90 platform description file provided by Affymetrix©. The final result of this work, data corrected for the artifact, has been created for 1494 samples. Affymetrix Yeast2 Array was based on 670 samples of GPL2529 GEO platform. Two mouse arrays were also analyzed: Affymetrix MOE430A and MOE430B. The analysis was based on 4277 data sets of the GPL339 platform for the Affymetrix MOE430A and 959 data sets for the GPL340 platform for the Affymetrix MOE430B array.

### Computing the correlations on a GPU

The positional artifact was detected through calculating the average correlation between probes at a given separation on the chip. These data sets were first normalized as the different samples come from many different experiments in a wide range of conditions. To this end we scaled the expression values in each sample by dividing them by the sample mean.

To calculate the average correlation between probes at a given separation on the chip, we calculated the Pearson correlation coefficient of the expression values across 1,383 arrays for all pairs of genes, and then computed the arithmetic mean for all pairs of probes with each given chip separation (dx, dy). When characterizing the positional artifact, we omitted genes with both neighboring chromosomal and neighboring chip locations, as these often share a regulatory sequence.

Repeatedly computing the correlation coefficients for all pairs of probes over many experiments is CPU-intensive. However, this problem is suitable for parallelization and scales very well on a large number of computing cores. Rather than parallelizing these calculations on a cluster of CPUs, we opted to run our calculations on a single graphics processing unit (GPU). The computational power of GPUs has surpassed that of CPUs and they come with a large number of cores at a much lower price per core. All cores on the GPU share one global memory, which also cuts the communication time between them significantly.

We used the CUDA (Compute Unified Device Architecture) programming interface from NVIDIA. In this work, we executed our algorithm on the NVIDIA GeForce GTX 275 that has 30 multiprocessors with 240 computing cores and 869 MB of memory. The most time-consuming step in our approach is the repeated calculation of the correlation coefficients between all probe-sets. One such cycle takes typically about 80 minutes on a single Intel® Xeon® W3540 CPU core, and has been a bottleneck in our optimization algorithm. The same calculation can be performed in approximately 10 seconds on the GeForce GTX 275 GPU using CUDA programming scheme (in single precision).

### Modeling the artifact and deconvolution of microarray data

We propose that the artifact results from a process in which a fraction of the signal overflows to other probes on the microarray, and can be described mathematically as convolution. Thus, the observed expression value, *E_o_*, for each probe is expressed as
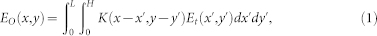
where (*x*,*y*) is the position of the probe and *K* is a kernel function that convolves the true expression value, *E_t_*. *L* and *H* denote the dimensions of the chip.

The kernel, *K*, consists of two components: a Dirac delta corresponding to the signal remaining in its original position and a Gaussian component describing the diffusion effect: The integration assumes *E_t_* = 0 for (*x′*,*y*′) outside of the chip.


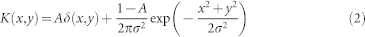
Here, *A* is the density amplitude of the Delta function (corresponding to the fraction of unaffected signal) and σ denotes the width of the Gaussian distribution. In practice, we use measurements binned onto pixels on the chip, corresponding to integer values of the position coordinates *x* and *y*. In this case, the Dirac delta is defined as δ(*x*,*y*) = 1 for (*x*,*y*) = (0,0) and δ(*x*,*y*) = 0 elsewhere. We optimized the shape of *K* in order to find such values of the parameters *A* and σ, for which deconvolution will remove the positional artifact without destroying the original data.

Deconvolution using the model given in Equation (1) is applied to *E_o_*(*x,y*) which is obtained for each sample by converting the expression values into a two-dimensional spatial matrix. The positions of the individual probes were used as indices for such matrix where each matrix has 285156 entries (number of probes). The deconvolution was performed in Fourier space and implemented on the GPU using the Fast Fourier Transform. The deconvolution process was applied to all 1383 samples. Finally, the corrected expression levels were rescaled to preserve their original distribution.

We optimized the parameters of the kernel function in order to obtain a possibly flat average correlation map. The average correlation between probes separated by a vector (*x*,*y*) in chip coordinates is defined as:
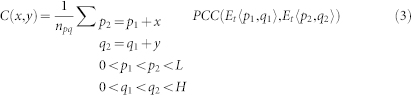
Here, *n_pq_* is the number of probe pairs (*p_1_*,*q_1_*), (*p_2_*,*q_2_*) with a given separation and *PCC*() is the Pearson correlation coefficient between deconvolved signal at two positions, computed over 1383 experiments. We characterized the flatness of the correlation map using an objective function based on the square of the first spatial moment of the average correlation:
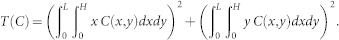


We optimized the parameters of *K* numerically by minimizing *T* using the Powell method. In each step, first the observed expression measurements in each of the 1383 samples used were deconvolved as described above. Next, correlation coefficients between all probe sets were calculated, and finally the average correlation map as function of probe separation was used to evaluate the objective function *T*. As a result of optimization, we obtained the following parameter values:
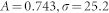
Using these kernel parameters, we obtained the final corrected expression values *E_o_*(*x,y*) for all experiments available in the GEO database.
